# Shame

**DOI:** 10.1007/s40037-018-0429-6

**Published:** 2018-04-23

**Authors:** Gretchen A. Case, Karly A. Pippitt, Benjamin R. Lewis

**Affiliations:** 1 Division of Medical Ethics and Humanities, Department of Internal Medicine, University of Utah School of Medicine, Salt Lake City, USA; 2 Department of Family and Preventive Medicine, University of Utah, Salt Lake City, USA; 3 Department of Psychiatry, University of Utah, Salt Lake City, USA; 4 Wakari Hospital, Dunedin, New Zealand

**Keywords:** Shame, Mistakes, Medical education, Pre-clinical education, Ethics, Humanities

## The story

We teach a required course for medical students that addresses aspects of medical practice that are hard to assess and make concrete, including professionalism, communication, ethics, humanities, and culture. Because this was the first iteration of a course created around issues that had been proven difficult to address rigorously in our curriculum, we designed the course around broad concepts that could provoke discussion on many fronts. In a session for approximately 80 second-year medical students, we explored the concept of shame and its possible manifestations in, and effects on, clinical medicine. For this first version of the course, we had the luxury and challenge of unusually long, multi-hour sessions, so we planned to address many variations of shame as it might manifest in either the patient or the provider role. Preparation for this session included readings from both professional and popular publications intended to offer several definitions of shame for consideration.

Given the potential for evoking highly emotional content we decided to begin our large-group session with a fairly benign depiction of shame. We drew on the ubiquitous internet meme of ‘dog shaming’, in which social media users post photographs of their dogs with a sign around their necks describing their bad behaviour, often with evidence (e. g., destroyed furniture) appearing in the background. The experience of shame here is externalized and anthropomorphized: the animals are not experiencing shame in a human way, but the humour of the photos allows entry to this difficult topic. We asked students to create their own ‘shaming photos’. To model the activity and build common ground, we three held up signs with our own mildly embarrassing secrets; for example, a faculty threw away a pizza in a box, and later took it out of the trash can and ate it. Students and faculty took photos of themselves holding their signs, all showing a clearly humorous, self-deprecating approach, like the dog-shaming meme we were mimicking. We projected these photos on a screen at the front of the lecture hall, and they were met with shared laughter.

Students then moved into small groups, facilitated by faculty (not including the authors), where they were tasked with creating a ‘shame scale’ modelled on the universal pain scales found in hospitals and clinics. After working together for about 45 minutes, the groups returned to the lecture hall to share their shame scales. As members of each group read their scale aloud in turn, we learned that some groups took the assignment quite lightly. Memorably, one group ranked Starbucks drinks from most to least shameful, clearly a tongue-in-cheek response to the assignment. But most groups delved more deeply and created scales reflecting levels of shame experienced by medical students. Listed as extremely shameful on more than one of these scales were ‘failing a course’ and ‘repeating a year in medical school’.

## Surprising outcome

Two students who were, in fact, remediating the year, quickly left the room before a full discussion of the scales was possible. When class ended, a contingent of students went directly to the administration offices to report that our course had publicly shamed students. They were particularly upset at the mention of remediation, as a student’s move from one cohort to another usually is more visible than the failure of one exam or one course. The students’ distress was evident enough that one of the authors was paged by administrators to discuss these concerns immediately. After discussion, the relevant Dean determined that no mistreatment had occurred and no further official action by that office was necessary. However, as course directors, we knew that this would not, and should not, be the end of the event.

Notably, the reaction here was not motivated by first-person offence but by indignation surrounding the *anticipated* offence to peers. It is telling, then, that when we reached out to the few students who were repeating the year, out of concern that they had experienced mistreatment or harm from the session, their reactions were quite different than those of their classmates. One student emailed back that, while upset in the moment, a short break from the intensity of the classroom was all that he/she needed to restore equanimity. This student did not report feeling a lasting sense of shame because of remediation nor because of the classroom discussion. Another student went into more depth in his/her email response. Again, this student did not feel shame about remediating and did not feel shamed by hearing the reading of the scales. This student was confident that remediation was the right choice for a complex personal situation. But he/she was deeply disappointed at the realization that fellow students assumed that shame would be the obvious and only emotion felt by a remediating student.

So while the classroom discussion of remediation as potentially shameful was construed, by upset, non-remediating students, as an act of course directors shaming remediating students, their attempts to protect classmates by appealing to higher authorities emphasized the perception of remediation as shameful. With an action meant to defend their classmates from shame, they shamed at least one of them. What we do not know, and probably will never know, is how many students felt shamed by other designations listed on the shame scales.

## Lessons learned

Shame is distinct from both *guilt* and *humiliation* in terms of perceived source, subjective character, and duration. In contrast to the temporary and local nature of guilt or humiliation (‘I did something wrong/bad’), shame implicates globally: the perceived insufficiency or flaws of the self are intrinsic, deep, and often significantly guarded (‘I am inherently wrong/bad’). In analyzing what happened, we find useful the following exposition: the experience of shame can be understood as the interaction of three factors: 1) the shame-inducing event, 2) the vulnerability of the subject, and 3) the social context [1]. Ironically, in this situation, the shame-inducing event was the exploration of shame itself.

The shame-inducing event was complex. There was first-order anticipated shaming of students currently remediating. However, we have no evidence of shaming from our subsequent interaction with these students. The other student we contacted echoed their colleague’s sentiment that remediation was the right choice for that situation. There was a second-order shaming of peers for the ways in which they had constructed shame scales: in this sense, students felt indignation towards the perceived attitudes and biases of peers. Finally, there was a third-order shaming of course instructors for not providing an adequate holding environment for this discussion and content: in not sufficiently controlling this content delivery per student estimations, we then had to respond to concerns that this exercise was unprofessional and possibly abusive on our part. Notably, the shaming involved did not occur within the characteristic rigid power hierarchy typically invoked in discussions of shame experiences within medical education (i. e., supervisor to supervisee) [2, 3]. Instead, the shaming was peer-to-peer and then up the chain of power towards instructors who were ultimately shamed by having to explain and reconcile these issues with the office of student affairs.

In this sense, the vulnerability of the subject is also complex. The vulnerability that presented itself during this exercise was not aligned with typical power dynamics characteristic of oft-criticized medical hierarchy. Rather, there was an *anticipated* vulnerability towards members of the student body (who themselves interestingly were relatively unperturbed by the activity) that provoked protective outrage.

Recent research [4] that describes how micro-aggressions and trigger warnings might affect the educational experience offers a possibly useful social context for further examination of our imbroglio. While this set of issues has received increasing attention in undergraduate education in recent years, we are not aware of any medical education literature that examines how this might complicate preclinical and clinical training. This is especially interesting to consider in light of the transition from the relatively familiar arena of classroom work to the unpredictable and volatile world of clinical medicine. An important hypothesis to explore is that as current cohorts of learners make their way through the existing structure of medical education, there will be increasing tensions between the emotional and moral expectations of learners and the instantiated historical model of training.

That the emotional intensity of this educational activity escalated so quickly demonstrates that sensitivity towards ‘shaming’ experiences is robustly active in medical education, even prior to the clinical years where it is generally characterized. Existing literature on medical education addresses the experience of shame primarily in the context of clinical training and practice, focusing on hierarchical issues of power and response to medical errors [5–7]. This literature focuses on the aggressive evaluative aspects of medical education as well as what has been termed the ‘hidden curriculum’. The experience of shame in the context of medical school has been linked to increasing rates of demoralization, burnout, and declining empathy among medical students progressing through their clinical rotations. The experience of shame is also relevant in examining and understanding the response of individual practitioners, as well as the profession, to medical errors. Here there is a clear connection between experienced shame and physician burnout, mental health, and suicide. Insofar as these issues are addressed in medical education, the focus is on the clinical years of training during the last two years of medical school and then in residency.

This activity and its aftermath offered us painful but valuable lessons. Breaking out into small groups was a useful way for students to explore this topic in a guided fashion. However, when the students returned to the large group, the context of each of these discussions was lost, and thus left intent and meaning open to wide interpretation. In this sense, small groups are clearly advantageous as the subject matter becomes increasingly nuanced. Larger groups also open the door to more reactive and socialized responses of vindictive protectiveness that may be unproductive for ongoing thoughtful discussion. The humorous scales stood in stark contrast to the more serious scales, and undermined useful discussion in a large group. Notoriously difficult to anticipate in its effects, humour provided a fulcrum here for moralizing reactions in a larger group setting where the individual interpersonal nuances were lost. The construction of ‘shame scales’ also implies objective evaluation as to the legitimacy of subjective emotional responses to shaming events, possibly invalidating those very responses. More focused instructions for facilitators and students, asking them to attend to the actual feeling of shame and not perceived shame or objective ratings of shame might have mitigated some of these issues. Examples of shame scales, further clarifying our expectations for the exercise, would also have been helpful. It is very clear that any discussion of shame in medical education and practice needs to be thoughtfully framed.

As medical educators, we know that the very fact that we stumbled into such treacherous ground when discussing shame means that we cannot turn away from this topic. Our initial inspiration to address shame broadly and creatively reinforced our thinking that many important events in the classroom and clinic require devoted attention to the discussion of shame. At the same time, we now have a painfully broadened perspective on how such discussions may be perceived by learners and what our responsibilities are as teachers, with considerable power, for mitigating harm. As further evidence of the complexity of the task, even the authors of this paper, who worked extremely well together as a teaching team, have disagreed on how that session could have been managed better. We have discussed the merits of our framing and instructions for the shame scale exercise, and argued about what outcomes we could have predicted. We have reflected on whether educators facing a failure like this one will shy away from important discussions about medical education and perhaps simplify the complexities of the medical work environment. We have considered the possibility of burgeoning tensions between the expectations and emotional needs of medical students and established styles of medical education that will continue to evolve in unexpected ways. However, we agree that this experience provides even stronger justification for the kind of material covered in the medical humanities and medical ethics as it can at least potentially provide a space for examination and understanding of these tensions. There is clearly a premium on doing this sensitively and thoughtfully given our experiences.

## Moral of the story

The reverberations of this one session, including our conversations with individual students, have deeply affected the way we teach this course since this incident. We learned a number of important lessons from this session about properly framing an activity and managing student expectations and reactions. More importantly, however, we learned how prevalent shame is in the pre-clinical years. We are convinced of the importance of *discussing shame without inducing shame *and supporting our colleagues in this work.
